# Supplementary figures and images for: Comparative study between radiofrequency-induced and muscimol-induced inhibition of cultured networks of cortical neuron
Source: PLoS One. 2022 Aug 31;17(8):e0268605. doi: 10.1371/journal.pone.0268605 (PMC9432733; doi:10.1371/journal.pone.0268605)

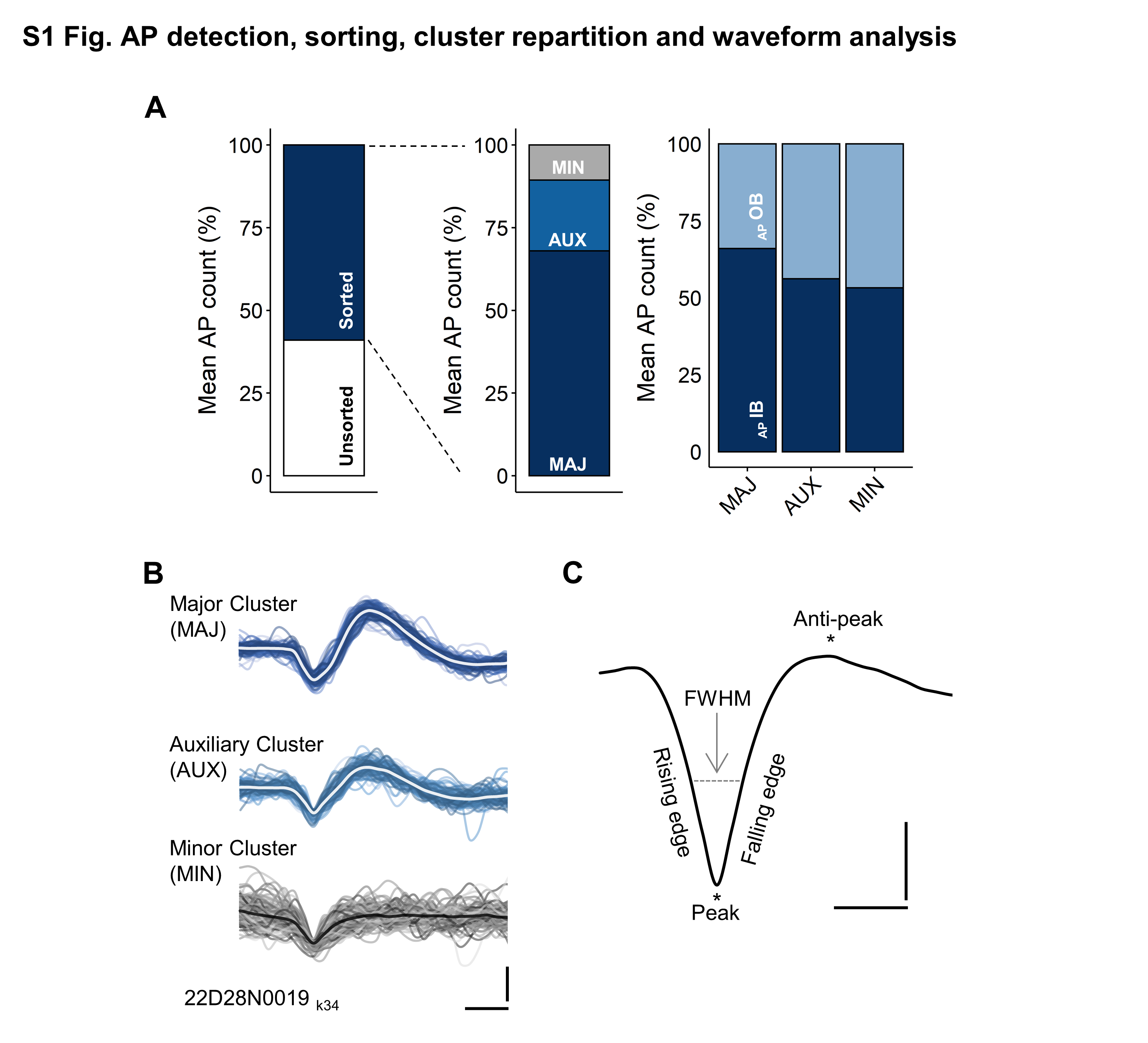

Supplement: S1 Fig — (A) From left to right: Mean AP detection for unsorted and sorted AP fraction (% of total detected APs); Relative fraction of sorted APs attributed to Major (MAJ), Auxiliary (AUX) and Minors (MIN) clusters; Mean AP count for sorted AP occurring either inside (AP IB) or outside (AP OB) bursts period. Data collected over 15 min during the pre-exposure phase from 15 cultures of the RF group used here as representative. (B) Example of sorted AP waveforms after principal component analysis and hierarchical classification, overlay of 125 waveforms per cluster with averaged waveform highlighted, data from one channel of the same culture. Scale: (y): 40 μV; (x): 500 μs. (C) Illustration of the metrics used to quantify changes in AP waveforms. FWHM: full width at half maximum. As recorded extracellularly the AP waveform is inverted. Scale: (y): 10 μV; (x): 500 μs. (TIF) [file pone.0268605.s001.tif]

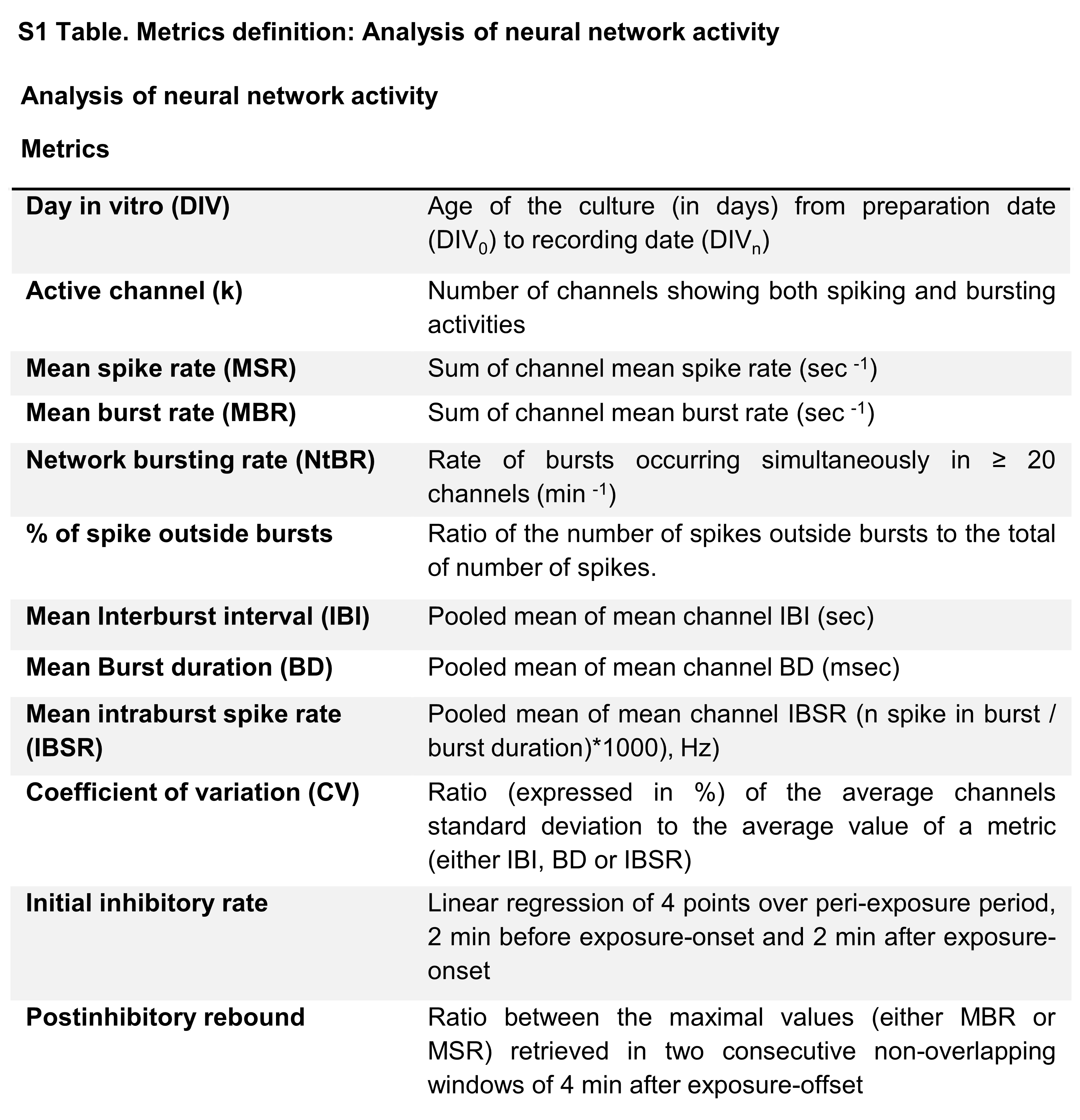

Supplement: S1 Table — (TIF) [file pone.0268605.s003.tif]

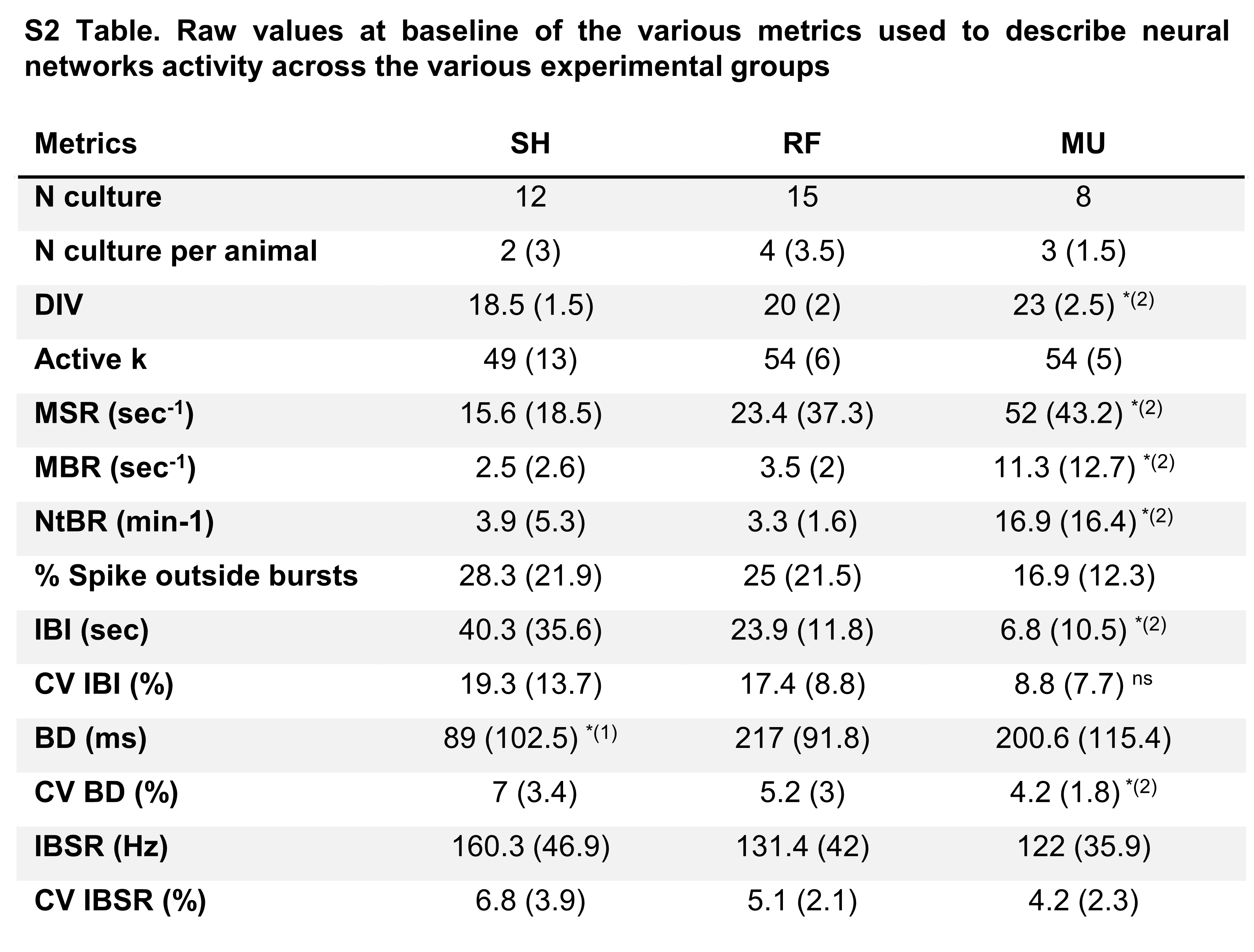

Supplement: S2 Table — Data expressed as Median (IQR). *(1) Indicates significant difference between SH-RF and SH-MU pairs (p < 0.05) and *(2) indicates significant difference between MU-SH and MU-RF pairs (p < 0.05), ns indicates no significant differences between groups. Pairwise comparison done with Kruskal-Wallis test followed by Conover’s all-pairs posthoc test. SH, n = 12; RF, n = 15; MU, n = 8. (TIF) [file pone.0268605.s004.tif]

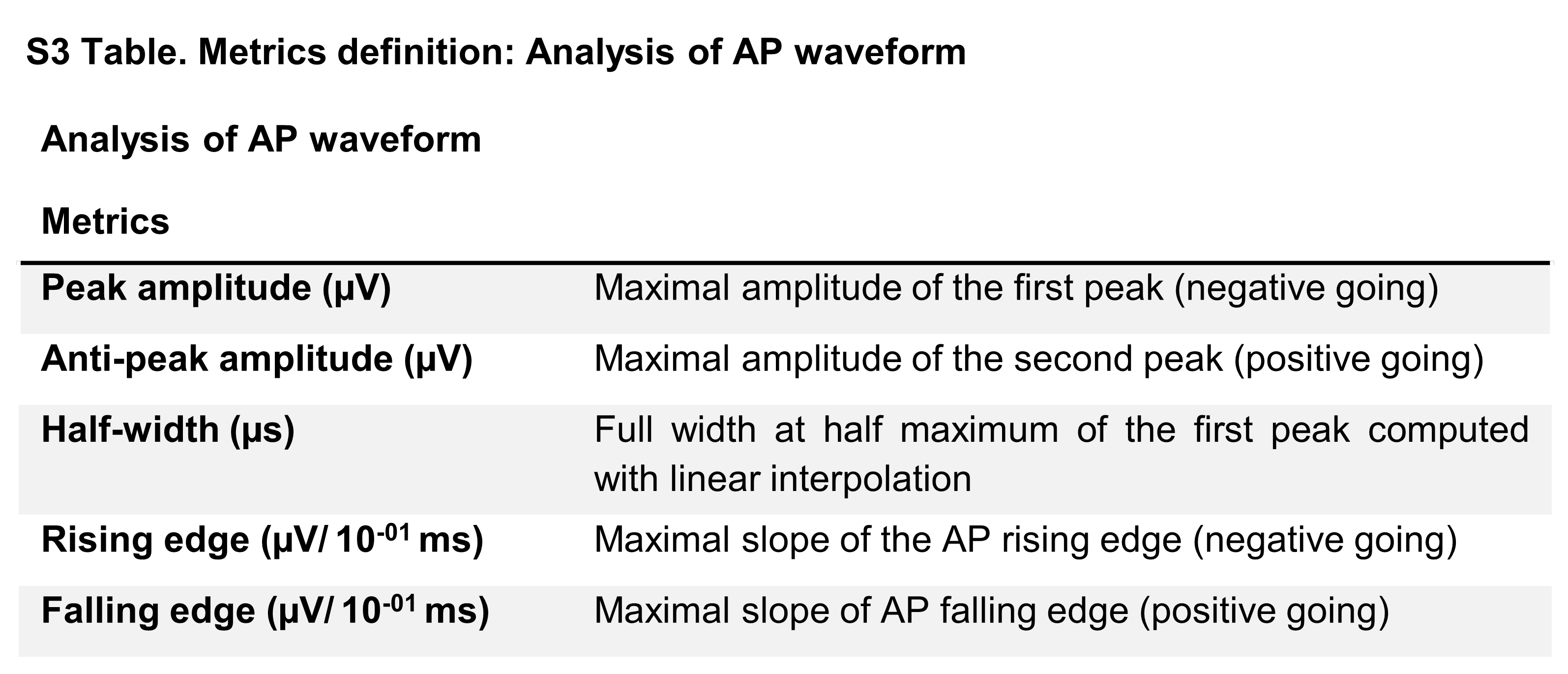

Supplement: S3 Table — (TIF) [file pone.0268605.s005.tif]

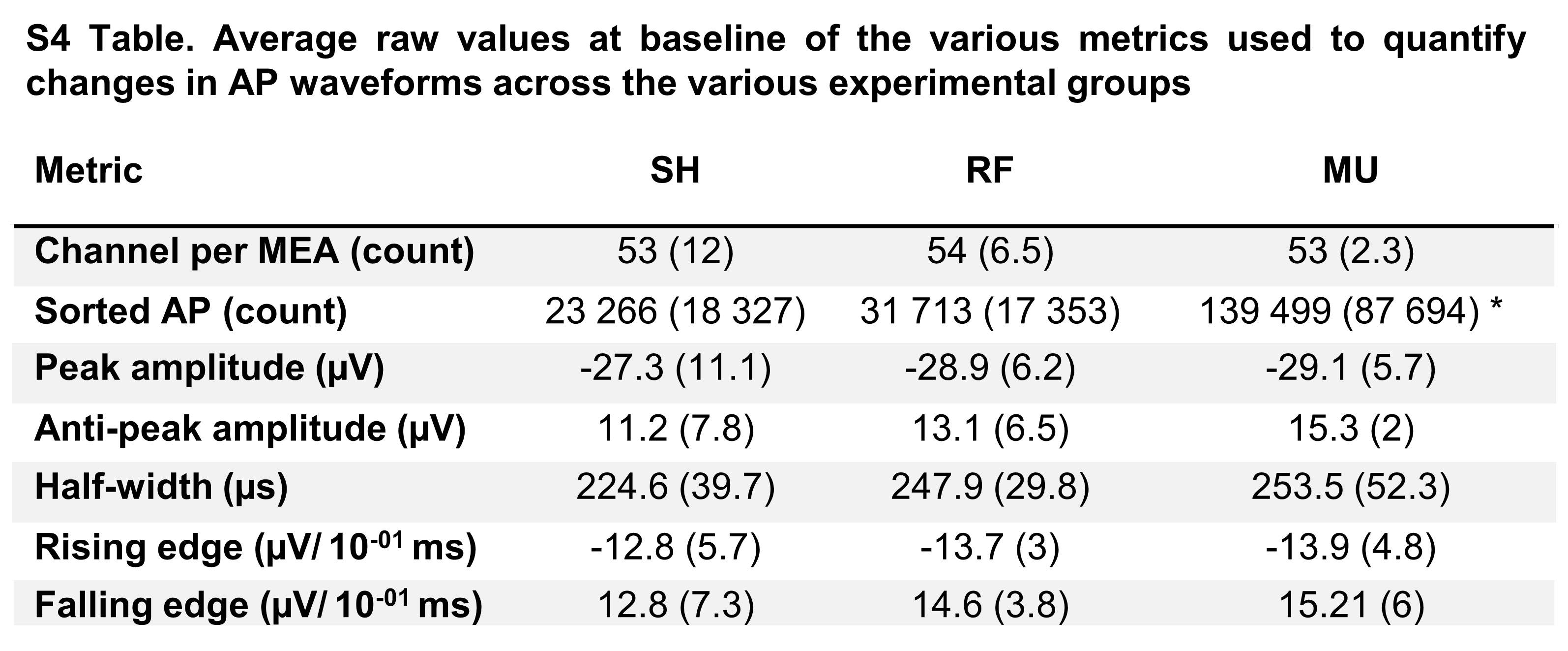

Supplement: S4 Table — Data expressed as Median (IQR). * Indicates significant difference (p < 0.05) between MU-SH and MU-RF pairs. Pairwise comparison done with Kruskal-Wallis test followed by Conover’s all-pairs posthoc test. SH, n = 12; RF, n = 15; MU, n = 8. (TIF) [file pone.0268605.s006.tif]
